# A novel system for predicting the toxicity of irinotecan based on statistical pattern recognition with *UGT1A* genotypes

**DOI:** 10.3892/ijo.2014.2556

**Published:** 2014-07-22

**Authors:** RYOUICHI TSUNEDOMI, SHOICHI HAZAMA, YUSUKE FUJITA, NAOKO OKAYAMA, SHINSUKE KANEKIYO, YUKA INOUE, SHIGEFUMI YOSHINO, TAKAHIRO YAMASAKI, YUTA KA SUEHIRO, KOJI OBA, HIDEYUKI MISHIMA, JUNICHI SAKAMOTO, YOSHIHIKO HAMAMOTO, MASAKI OKA

**Affiliations:** 1 Department of Digestive Surgery and Surgical Oncology, Yamaguchi University Graduate School of Medicine, Yamaguchi 755-8505, Japan; 2 Department of Computer Science and Systems Engineering, Faculty of Engineering, Yamaguchi University, Yamaguchi 755-8611, Japan; 3 Department of Clinical Laboratory, Yamaguchi University Hospital, Yamaguchi 755-8505, Japan; 4 Translational Research and Clinical Trial Center, Hokkaido University Hospital, Sapporo 060-8638, Japan; 5 Unit of Cancer Center, Aichi Medical University, Nagakute 480-1195, Japan; 6 Tokai Central Hospital, Aichi 504-8601, Japan

**Keywords:** irinotecan, polymorphisms, prediction, toxicity, uridin diphosphate-glucuronosyltransferase 1A

## Abstract

To predict precisely severe toxicity of irinotecan, we evaluated the association of *UGT1A* variants, haplotypes and the combination of *UGT1A* genotypes to severe toxicity of irinotecan. UGT1A1*6 (*211G*>*A*), UGT1A1*28 (*TA_6_*>*TA_7_*), UGT1A1*60 (−*3279T*>*G*), *UGT1A7* (*387T*>*G*), *UGT1A7* (*622T*>*C*), and UGT1A9*1b (−*118T_9_*>*T_10_*_,_ also named *22) were genotyped in 123 patients with metastatic colorectal cancer who had received irinotecan-based chemotherapy. Among the 123 patients, 73 were enrolled in either of two phase II studies of the FOLFIRI (leucovorin, 5-fluorouracil and irinotecan) regimen; these patients constituted the training population, which was used to construct the predicting system. The other 50 patients constituted the validation population; these 50 patients either had participated in a phase II study of irinotecan/5′-deoxy-5-fluorouridine or were among consecutive patients who received FOLFIRI therapy. This prediction system used sequential forward floating selection based on statistical pattern recognition using *UGT1A* genotypes, gender and age. Several *UGT1A* genotypes [UGT1A1*6, *UGT1A7* (*387T*>*G*), *UGT1A7* (*622T*>*C*) and UGT1A9*1b] were associated with the irinotecan toxicity. Among the haplotypes, haplotype-I (*UGT1A1*: −3279T, TA_6_, 211G; *UGT1A7*: 387T, 622T; *UGT1A9*: T_10_) and haplotype-II (*UGT1A1*: −3279T, TA_6_, 211A; *UGT1A7*: 387G, 622C; *UGT1A9*: T_9_) were also associated with irinotecan toxicity. Furthermore, our new system for predicting the risk of irinotecan toxicity was 83.9% accurate with the training population and 72.1% accurate with the validation population. Our novel prediction system using statistical pattern recognition depend on genotypes in *UGT1A*, age and gender; moreover, it showed high predictive performance even though the treatment regimens differed among the training and validation patients.

## Introduction

Concurrent irinotecan and fluorinated-pyrimidine is a common first-line therapy for metastatic colorectal cancer (mCRC) (1–6). Although prolonged survival is associated with regimens involving irinotecan, severe neutropenia occurs in 20–35% of mCRC cases treated with irinotecan regimens. Carboxylesterases catabolized irinotecan to 7-ethyl-10-hydroxycamptothecin (SN-38), which is a potent topoisomerase I inhibitor (7,8). SN-38 is then further catabolized by hepatic uridin diphosphate-glucuronosyltransferase (UGT) 1A enzymes to an inactive SN-38 glucuronide (SN-38G) (9). Many mCRC patients with a genetic variant (UGT1A1*28) experience severe irinotecan toxicity; UGT1A1*28 is a variation in the number (seven vs. six) of TA repeats in the promoter region of *UGT1A1* (10,11). Interestingly, the toxicity and tumor response of concurrent leucovorin, 5-fluorouracil, and irinotecan (FOLFIRI) reportedly also correlate with *UGT1A* variants (*UGT1A1*, *UGT1A7* and *UGT1A9*) and haplotypes including these variants (12–18). There are differences between Caucasian and Asian populations in frequencies of *UGT1A* variants, and UGT1A1*6 reportedly associates strongly with severe neutropenia especially among Asian patients (12,17).

To predict the risk of irinotecan toxicity for individual patients, it is important that determining the relative contributions of *UGT1A* variants other than UGT1A1*28 and UGT1A1*6 is important to the development of any system designed to predict irinotecan toxicity for individual patients because patients without UGT1A1*28 or *6 do experience severe irinotecan toxicity. Several studies have examined associations between irinotecan toxicity and *UGT1A* haplotypes in addition to each genotype of *UGT1A* (17–19). However, determining the haplotype or diplotype for each patient is difficult; moreover, most haplotypes and diplotypes are too rare to constitute a group large enough for meaningful statistical analysis. Moreover, gender and age of patients each reportedly have an impact on irinotecan toxicity (20–22). Hence, these factors should be also taken into consideration when developing a system designed to predict irinotecan toxicity.

The aim of this study was to evaluate whether the combinations of *UGT1A* genotypes, but not haplotypes, together with patient characteristics might be useful in predicting the risk to patients with mCRC treated of irinotecan-containing regimens. Here, we investigated the genotypes of 123 patients at six loci: UGT1A1*6 (211G>A, rs4148323), UGT1A1*28 (TA_6_>TA_7_, rs8175347), UGT1A1*60 (−3279T>G, rs4124874), *UGT1A7* (387T>G, rs17868323), *UGT1A7* (622T>C, rs11692021), and UGT1A9*1b (−118T_9_>T_10_, rs35426722, also called UGT1A9*22) (23). Next, we evaluated the contribution of each *UGT1A* genotype, haplotype, and diplotype to the risk of irinotecan toxicity. Furthermore, we developed a new system for predicting the risk that a patient will experience irinotecan toxicity; this system uses sequential forward floating selection (SFFS) algorithm based on statistical pattern recognition to select the combinations of *UGT1A* genotypes, gender and age. SFFS is a sequential search method characterized by a dynamically changing number of features included or eliminated at each step of an individual analysis (24). This is the first study conducted to assess the role of the combination of genotypes at six polymorphic sites in *UGT1A* and clinical features constructed by SFFS on the risk of irinotecan toxicity.

## Materials and methods

### Patients

In this study, 123 mCRC patients were examined for association between *UGT1A* genotypes and irinotecan toxicity (Table I). This study was performed as an ancillary investigation; data collected from three prospective studies [FLIGHT1 (5), FLIGHT2 (5) and FRUTIRI (6)] and from consecutive patients who received FOLFIRI at the Department of Digestive Surgery and Surgical Oncology, Yamaguchi University Graduate School of Medicine, Japan. Each participant received irinotecan at the dose of 150 mg/m^2^, which has been approved in Japan.

FLIGHT1 (UMIN000002388) and FLIGHT2 (UMIN000002476) were phase II studies of first line and second line chemotherapy, respectively, for mCRC. Study designs and key eligibility and exclusion criteria have been described in detail (5,25,26). Briefly, each regimen consisted of irinotecan on day 1 +400 mg/m^2^ fluorouracil bolus followed by 2,400 mg/m^2^ fluorouracil continuous infusion during 46 h + 200 mg/m^2^ leucovorin on day 1 every 2 weeks. Of all patients from the FLIGHT1 and FLIGHT2 studies, 38 and 35, respectively, participated in this ancillary investigation and use; these 73 patients constituted the training population. FLIGHT1 or FLIGHT2 patients homozygous for UGT1A1*28 were excluded from the training population because these patients received a lower starting dose of irinotecan (100 mg/m^2^) (5).

The validation population comprised 50 patients from two different study groups: 22 patients who participated in FRUTIRI (UMIN000005011), a phase II study of a combination therapy comprised irinotecan and 5′-deoxy-5-fluorouridine (5′-DFUR) (6) and 28 consecutive patients who underwent second-line FOLFILI treatment between October, 2008 and July, 2012 in the Department of Digestive Surgery and Surgical Oncology, Yamaguchi University Graduate School of Medicine, Japan. Detail treatment regimen tested in FRUTIRI was described previously (6). Briefly, irinotecan was administered every two weeks, and 400 mg 5′-DFUR was administered every week orally twice a day on five consecutive days that were followed by a weekly 2-day washout. The 28 consecutive patients undergoing FOLFIRI treatment were following the protocol used in FLIGHT2 (26). In a validation population, patients with UGT1A1*28 homozygous were not found in the FRUTIRI study (n=28). Additionally, patients heterozygous for UGT1A1*28 (n=6) were excluded from the FRUTIRI study because these patients received lower starting dose of irinotecan 70 mg/m^2^. Among the 28 consecutive patients who received second-line FOLFILI therapy, homozygous for UGT1A1*6 or *28 and those compound heterozygous for UGT1A1*6 and UGT1A1*28 been excluded from this ancillary study. The training (n=73) and validation (n=50) populations did not differ significantly with regard to the distribution of any clinical feature or genotype that is listed in Table I except for the distributions of the *UGT1A7* (*387T*>*G*) and UGT1A9*1b alleles (data not shown).

In this study, we defined patients who exhibited hematologic toxicity greater than grade 3 during the entire course of therapy as experiencing irinotecan toxicity. The study protocols were approved by the Institutional Review Board at Yamaguchi University Graduate School of Medicine, and were carried out in accordance with the Helsinki declaration on experimentation on human subjects. Each patient gave written, informed consent for their participation in this study.

### Genotyping of UGT1A and haplotype construction

A conventional sodium iodide (NaI) method was used to extract genomic DNA from peripheral blood samples (27). The number of TA repeats in the *UGT1A1* promoter region was determined by the fragment size analysis followed by direct sequencing as described previously (4). The TaqMan technique with a hydrolysis probe was used to determine the UGT1A1*6 genotype as described previously (28); similarly, hydrolysis probes were used to determine the genotypes at UGT1A1*60; a direct sequencing method was also used to determine the genotypes at *UGT1A7* (387T>G and 622T>C) and UGT1A9*1b.

Each nucleotide variant was evaluated to determine whether it was in Hardy-Weinberg equilibrium; Haploview 4.2 software was used to perform the linkage disequilibrium (LD) and case-control haplotype analyses (29). Lewontin’s coefficient D’ and correlation coefficient *r*^2^ were calculated as measures of LD.

### Construction of toxicity prediction system by genotype combinations

To predict severe toxicities of irinotecan, the age, the gender and a comprehensive 6-site *UGT1A* genotype were determined for each of the 73 patients in the training population. SFFS, a method of statistical pattern recognition, was then used to determine the optimal genotype combinations for predicting the risk of irinotecan toxicity. The statistical pattern recognition, SFFS, identified the genotype combinations with the ‘maximum number of cases’ and ‘maximum prediction rate’ to maximize overall diagnostic accuracy (24). Briefly, the algorithm of the SFFS used in this study was as follows: i) Suppose that at stage *k* we have a set of *X_1_*, …, *X_k_* of sizes 1 to *k*, respectively. ii) Let the corresponding values of the feature selection criteria be *J_1_* to *J_k_*, where *J_i_* = *J*(*X_i_*), for the feature selection criterion *J*(.). iii) Let the total set of features be *X*. Then at the *k*th stage of the SFFS procedure follow these steps: Step 1, select the feature *x_j_* from *X*-*X_k_* that increases the value of *J* to the greatest degree and add it to the current set: *X*_(_*_k_*_+ 1)_ = *X_k_* + *x_j_*. Step 2, find the feature *x_r_* in the current set *X*_(_*_k_*_+ 1)_ that reduces the value of *J* the least; if this feature is the same as *x_j_* then set *J*_(_*_k_*_+ 1)_ = *J*(*X*_(_*_k_*_+ 1)_); increment *k*; go to step 1; otherwise remove it from the set to from *X*′*_k_* = *X*_(_*_k_*_+ 1)_ - *x_r_*. Step 3, continue removing features from the set *X*′*_k_* to form reduced sets *X*′ _(_*_k_*_− 1)_ while *J*(*X*′ _(_*_k_*_− 1)_) > *J*_(_*_k_*_− 1)_; *k* = *k* − 1; until *k* = 2; then continue with step 1. The algorithm is initialized by setting *k* = 0 and *X_0_* = Ø.

### Statistical analysis

Fisher’s exact test was used to assess the relationship between toxicity and each *UGT1A* variant. The Cochran-Armitage trend test was used to examine the linearity of the relationship between *UGT1A* genotypes and irinotecan toxicity. SPSS Statics 17.0 software (IBM, Tokyo, Japan) and R version 2.13.0 software were used to perform the calculations (30). p<0.05 was considered statistically significant.

## Results

### UGT1A allele and haplotype frequencies

**Figure 1:**
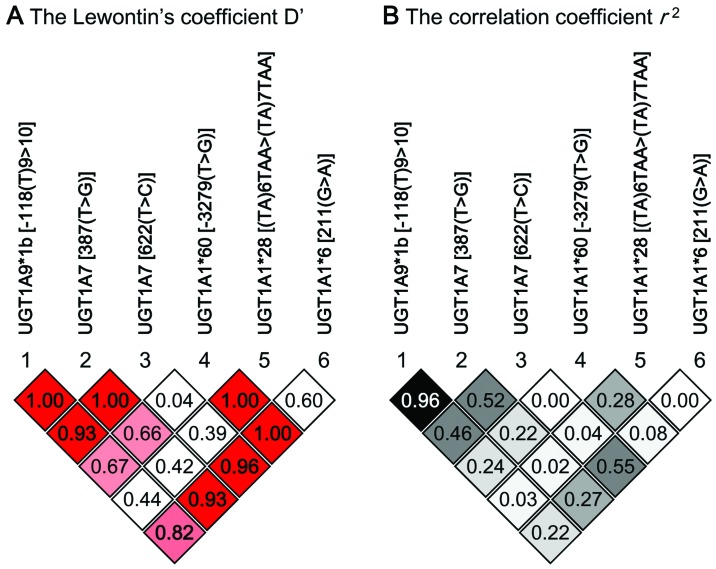
Pairwise linkage disequilibrium relationships between the *UGT1A* variants. (A) The Lewontin’s coefficient D′ and (B) the correlation coefficient *r*^2^ are represented as values and colors [in panel A, log of the odds (LOD) ≥2 shades of pink/red, LOD <2 and D′=1 is blue, and LOD <2 and D′ <1 is white. In panel B, *r*^2^<0.01 is white, 0.01≤*r*^2^ <0.95 is shades of grey, and *r*^2^≥0.95 is black] in each box.

The minor allele frequencies (MAF) of each *UGT1A* allele among the 103 patients without genetic bias; all patients regardless of the starting dose of irinotecan enrolled in FLIGHT1, FLIGHT2, and FRUTIRI studies, and 123 patients received a starting dose of 150 mg/m^2^ for case-control study participating in this study are listed in Table II. In this study, the MAFs of UGT1A1*28 and UGT1A1*6 were approximately 0.117 and 0.184, respectively. The MAF for each other *UGT1A* SNP examined in this study was greater than 0.20. Among all patients, the Hardy-Weinberg equilibrium p-value for each locus examined in this study was higher than 0.05. LD analysis with 103 patients showed that high LD (*r*^2^>0.9) was evident between *UGT1A7* (*387T*>*G*) and UGT1A9*1b (Fig. 1). We found 12 *UGT1A* haplotypes (*Hp-I* to *Hp-XII*) using 6 loci in 103 patients: UGT1A1*6, *28, *60, *UGT1A7* (*387T*>*G*), *UGT1A7* (*622T*>*C*), and UGT1A9*1b (Table III). Three common haplotypes (*Hp-I*, *Hp-II* and *Hp-III*) accounted for 82.5% of all haplotypes identified in this study.

### Associations between UGT1A genotypes/haplotypes and irinotecan toxicity

We examined associations between individual *UGT1A* genotypes or haplotypes and severe irinotecan toxicity among 123 patients with mCRC who receive chemotherapy that included irinotecan (Table IV). Each of four *UGT1A* genotypes [UGT1A1*6, *UGT1A7* (*387T*>*G*), *UGT1A7* (*622T*>*C*) and UGT1A9*1b] showed a significant association to irinotecan toxicity and linear trend (p<0.05). Similarly, two haplotypes (*Hp-I* and *Hp-II*) each showed a significant association to and linear trend with irinotecan toxicity (p<0.05). Among two patients received a starting dose of 100 mg/m^2^ irinotecan, diplotype of *Hp-IV/V* did not show toxicity and diplotype of *Hp-V/V* showed toxicity. Six patients excluded from FRUTIRI study did not show toxicity of irinotecan (a starting dose of 70 mg/m^2^; *UGT1A* diplotypes of *Hp-I/V*, *II/IV* and *III/IV* were found in 2, 3 and 1 patients). Regarding non-hematological toxicities, only 5 patients developed grade 3 diarrhea (*UGT1A* diplotype of these 5 patients consists of 4 *Hp-I/II* and 1 *Hp-II/XII*).

### Performances of the toxicity prediction system by genotype combination

**Figure 2:**
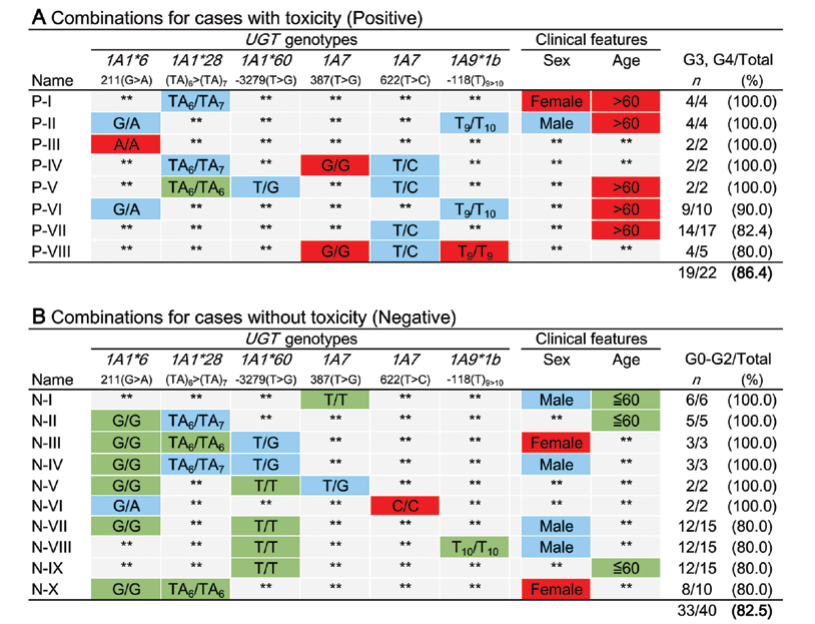
The *UGT1A* genotype combinations that predict the presence or absence of severe irinotecan toxicity based on statistical pattern recognition. (A) A total of 8 combinations (P-I to P-VIII) for positive prediction of the toxicity and (B) 10 combinations (N-I to N-X) for negative prediction are presented. (A) The 8 combinations (P-I to P-VIII) that predict the presence of irinotecan toxicity are shown. (B) The 10 combinations (N-I to N-X) that predict the absence of irinotecan toxicity are shown. Eight factors-patient age, patient gender and genotypes at six *UGT1A* sites [UGT1A1*6, *28, *60, *1A7* (*387T*>*G*), *1A7* (*622T*>*C*) and 1A9*1b] were used with sequential floating forward selection (SFFS) for statistical pattern recognition as described in Materials and methods. Homozygosity for alleles associated with irinotecan toxicity, heterozygosity and homozygosity for alleles not associated with irinotecan toxicity are indicated by red, blue and green cells, respectively. ^**^The un-specified categories (regardless of genotypes, gender or age).

To construct a system for predicting the risk of severe irinotecan toxicity, genetic data from 73 patients that constituted the training population were analyzed exhaustively; specifically, SFFS was used to assess gender, age and the individual genotypes at six polymorphic *UGT1A* sites (Fig. 2). In addition to the three possible genotypes (wild-type homozygous, heterozygous, variant homozygous), a fourth option for each site (designated ‘unspecified genotype’) was included into the algorithm. Similarly, patient gender (male, female, regardless of gender) and age (≤60, >60 years old, regardless of age) were assessed. The cutoff value for age (60 years) was determined by Youden index obtained by the receiver operating characteristic (ROC) curve analysis with the training population. Among possible combinations (4^6^ × 3^2^ − 1 = 36,863), the following cases were excluded: cases not found, single cases, and cases that represented positive or negative predictive values <80%. In order to optimize the combinations, categorization according to predictive value and exclusion of redundant combinations in each category were performed. As a result, 8 combinations (P-I to P-VIII, Fig. 1A) appeared to predict an increased risk of toxicity, and 10 combinations (N-I to N-X, Fig. 1B) appeared to predict a lack of toxicity.

The system for predicting irinotecan toxicity based on combinations of 8 factors (6 genotypes, gender and age) was generated using data from of all 73 patients in the training population. The system was then applied to data from 84.9 and 86.0% of the patients in the training and validation populations, respectively (Table V). This prediction system showed 83.9% accuracy (positive predictive value, 86.4%; negative predictive value, 82.5%) for the training population (n=62) and 72.1% accuracy (positive predictive value, 70.0%; negative predictive value, 72.7%) for the validation population (n=43). When patients who were not applied to the combinations were included, the performance of the system was 71.2% accuracy (sensitivity, 55.9%; specificity, 84.6%) in training population (n=73) and 62.0% accuracy (sensitivity, 41.2%; specificity, 72.7%) in validation population (n=50). Odds ratios of positive prediction for irinotecan toxicity for this prediction system were 8.0 (95% CI, 1.5–42.5) and 16.3 (95% CI, 2.2–121.4) in training and validation populations, respectively (p<0.05, Table VI).

Patients with either of three *UGT1A* alleles [UGT1A1*6, *UGT1A7* (*622T*>*C*) or UGT1A9*1b], *UGT1A haplotype-I* or *haplotype-II* showed significant association to severe irinotecan toxicity (p<0.05) in both the training and validation populations (data not shown).

## Discussion

The novel system for predicting severe irinotecan toxicity described here was based on genotypes at 6 polymorphic sites in *UGT1A* and 2 basic clinical features; notably, it showed high predictive performance even though the treatment regimens differed among the training and validation patients (Tables V and VI). The odds ratio of positive prediction for severe irinotecan toxicity was higher for this prediction system than for that of any other haplotype or for that of any genotype (Table VI). The performance of this prediction system was reduced from the 83.9% accuracy seen with applied patients to this system in the training population to 72.1% accuracy in the validation. With regard to positive prediction, the inconsistency in accuracy between training and validation populations was seen when the combinations included the UGT1A9*1b site and patient age (P-II, VI and VII in Fig. 2). The frequencies of UGT1A9*1b genotype differed between the training and validation populations; moreover, the UGT1A9*1b alleles were not in Hardy-Weinberg equilibrium in the validation population (data not shown). The cutoff value for patient age (60 years old) was determined by a ROC curve generated with data from the training population; however, previous studies used a cutoff age of 65 years (20,21). Indeed, one patient without toxicity, but predicted as presence of toxicity in this system, was aged 63 years.

Some genotypic combinations decreased the performance of negative prediction for sever irinotecan toxicity in the validation population relative to the training population (N-II, IV, and V in Fig. 2). Specifically, 36.4% (n=4/11) of patients in training population with a combined genotype that included heterozygous for UGT1A1*28 alleles and UGT1A1*6 (−*/*−) experienced severe irinotecan toxicity, but 66.7% (n=4/6) of the patients in validation population with the same genotype combinations (UGT1A1*6, −*/*− and UGT1A1*28, −/+) showed severe toxicity. Of the 73 patients in the training population and the 50 in the validation population, 11 (15.1%) and 7 (14.0%), respectively, were matched with neither of the combination in our prediction system. Interestingly, the incidence of severe toxicity among patients who were not matched with either combination identified by this prediction system was 72.7% (training population) and 14.3% (validation population) (Table VI). Therefore, the frequency of the irinotecan toxicity among patients who do not have any combination of *UGT1A* variants identified by this novel prediction system might be due to factors other than *UGT1A* polymorphisms.

Many published studies have focused on associations between irinotecan toxicity, irinotecan efficacy, or both and any one or more of each *UGT1A* variants examined here (10–19,31,32). Patients, especially Asian patients, homozygous for UGT1A1*6 or *28 or compound heterozygous for these variants are at high risk for hematologic toxicity (13,33,34). In this study, each patient homozygous for UGT1A1*6 (n=3) and those compound heterozygous for UGT1A1*6 and *28 (n=3) showed severe hematologic toxicity; however, 45 patients of the remaining 117 patients still exhibited severe irinotecan toxicity. UGT1A1*6 and *28 each have strong effects on UGT1A1 activity and expression, but frequency of each allele is low; moreover, the frequencies of each allele differ between races (11,14,35–37). Among the patients that lacked these rare, highly effective variants, this novel prediction system could accurately predict whether there is severe irinotecan toxicity.

Here, as in previous studies, each identified *UGT1A* haplotypes was useful for precisely predicting the presence or absence of severe irinotecan toxicity (14,18,38–40). Consistent with our study, Cecchin *et al* reported that a haplotype comprising UGT1A1*28 (−), UGT1A1*60 (−), *UGT1A7* (*387T* and *622T*), and UGT1A9*1b (+) was a predictor of severe hematologic toxicity during the entire course of therapy (18). However, determining the haplotypes for any one patient is a difficult clinical measurement. Therefore, the genotypes at each of the 6 sites (rather than the haplotype or diplotype) could be used for clinical assessments.

Our prediction system depend not only on *UGT1A* genotypes but also on patient gender and age. Previous studies showed that patient gender and age were related to the risk of irinotecan toxicity (20–22). In the training population, patient age was associated with severe irinotecan toxicity, but patient gender was not (Table IV). Interestingly, when patient age, patient gender or both the patient age and gender were excluded from the factors used by the prediction system, the number of patients that matched with the prediction system decreased, although the system maintained the high positive and negative predictive values (data not shown).

The SFFS algorithm could be modified to include other factors (e.g., mutations in the tumor, patients’ clinical characteristics, additional genetic variants, etc.) to improve the prediction performance. Such modifications may result in a system that could meaningfully predict clinical outcomes, including tumor response. Recent advances in technology for sequencing whole genomes of individuals may lead to substantial increases in information that might be useful for personalized therapy. However, such complicated information could not be efficiently or fully utilized in the currently available formats. SFFS could easily construct a system that can utilize huge data sets such as whole-genome sequences. Our strategy for developing SFFS-based systems for clinical use could serve as a powerful tool for advancing personalized therapy, although additional prospective study of this prediction system is needed.

## Figures and Tables

**Table I:** Characteristics of the patients.

		Sub-population (treatment regimen)				
			
Clinical features and genotypes	Total (n=123)	FLIGHT1^a^ (n=38)	FLIGHT2^a^ (n=35)	FRUTIRI^b^ (n=22)	2nd-line FOLFILI^c^ (n=28)	
Toxicity of irinotecan						
No	72	20	19	16	17	NS^d^
Yes	51	18	16	6	11	
Gender						
Male	78	24	24	17	13	NS^d^
Female	45	14	11	5	15	
Age						
≤60	50	14	14	9	13	NS^d^
>60	73	24	21	13	15	
UGT1A1*6						
−*/*−	84	25	23	15	21	NS^d^
−/*6	36	12	11	6	7	
*6/*6	3	1	1	1	0^c^	
UGT1A1*28						
−*/*−	103	32	27	22	22	NS^d^
−/*28	20	6	8	0^b^	6	
*28/*28	0	0^a^	0^a^	0^b^	0^c^	
UGT1A1*60						
−*/*−	71	19	21	15	16	NS^d^
−/*60	46	17	12	6	11	
*60/*60	6	2	2	1	1	
*UGT1A7*						
387T/T	41	13	12	8	8	NS^d^
387T/G	69	18	18	13	20	
387G/G	13	7	5	1	0	
*UGT1A7*						
387T/T	70	21	19	14	16	NS^d^
387T/G	48	15	13	8	12	
387G/G	5	2	3	0	0	
UGT1A9*1b						
*1b/*1b	43	14	12	9	8	NS^d^
−/*1b	67	17	18	12	20	
−*/*−	13	7	5	1	0	

The following patients were not enrolled in this study as described in Materials and methods.

a Patients bearing UGT1A1*28 homozygous were excluded from the FLIGHT1 and FLIGHT2 studies.

b Homozygous and heterozygous of UGT1A1*28 were not enrolled in the FRUTIRI study.

c Homozygous of UGT1A1*6 and *28 and compound heterozygous of UGT1A1*6 and *28 were not included in the consecutive patients received second-line FOLFILI therapy.

d NS, not significant among 4 groups by Fisher’s exact test.

**Table II:** Minor allele frequency and Hardy-Weinberg equilibrium in 123 patients.

	103 patients^a^		123 patients^b^	
		
	MAF	HWp	MAF	HWp
UGT1A1*6 [211 (G>A)]	0.18	1.00	0.17	1.00
UGT1A1*28 [(TA)_6_>(TA)_7_]	0.12	0.80	0.08	0.86
UGT1A1*60 [−3279 (T>G)]	0.27	0.99	0.24	0.92
*UGT1A7* [387 (T>G)]	0.42	1.00	0.39	0.07
*UGT1A7* [622 (T>C)]	0.27	0.61	0.24	0.54
UGT1A9*1b [−118 (T_9_>T_10_)]	0.41	0.84	0.38	0.13

a Patients enrolled in the FLIGHT1, FLIGHT2 and FRUTIRI studies (patients received lower starting dose of irinotecan were not excluded).

b Patients subjected to case-control study (patients received lower starting dose of irinotecan were excluded).

MAF, minor allele frequency. HWp, p-value of Hardy-Weinberg equilibrium.

**Table III:** Haplotype frequency.

Haplotypes	*UGT1A* alleles						Allele frequencies	
	
	*UGT1A9*	*UGT1A7*		*UGT1A1*			(n=103)^b^	(n=123)^c^
	*1b	387T>G	622T>C	*60	*28	*6		
*Hp-I*	T_10_	T	T	T	TA_6_	G	0.524	0.573
*Hp-II*	T_9_^a^	G^a^	C^a^	T	TA_6_	A^a^	0.170	0.159
*Hp-III*	T_9_^a^	G^a^	T	G^a^	TA_6_	G	0.131	0.134
*Hp-IV*	T_9_^a^	G^a^	C^a^	G^a^	TA_7_^a^	G	0.063	0.041
*Hp-V*	T_10_	T	T	G^a^	TA_7_^a^	G	0.044	0.028
*Hp-VI*	T_9_^a^	G^a^	C^a^	T	TA_6_	G	0.015	0.016
*Hp-VII*	T_9_^a^	G^a^	C^a^	G^a^	TA_6_	G	0.015	0.012
*Hp-VIII*	T_9_^a^	G^a^	T	G^a^	TA_7_^a^	G	0.010	0.012
*Hp-IX*	T_10_	T	T	G^a^	TA_6_	G	0.010	0.008
*Hp-X*	T_10_	G^a^	C^a^	T	TA_6_	A^a^	0.010	0.008
*Hp-XI*	T_9_^a^	G^a^	T	T	TA_6_	G	0.005	0.004
*Hp-XII*	T_10_	T	T	T	TA_6_	A^a^	0.005	0.004

a Association of the alleles with toxicity of irinotecan.

b Patients enrolled in the FLIGHT1, FLIGHT2 and FRUTIRI studies (patients received lower starting dose of irinotecan were not excluded).

c Patients subjected to the case-control study (patients received lower starting dose of irinotecan were excluded).

**Table IV:** Associations between *UGT1A* genotypes/haplotypes and irinotecan toxicity.

		Toxicity			p-value	
			
		Yes	No	(% of yes)	Fisher’s exact	CA trend
Genotypes						
UGT1A1*6	−*/*−	27	57	(32.1)	0.002	0.001
	−/*6	21	15	(58.3)		
	*6/*6	3	0	(100.0)		
UGT1A1*28	−*/*−	40	63	(38.8)	0.218	-
	−/1*28	11	9	(55.0)		
	1*28/1*28	-	-	-		
UGT1A1*60	−*/*−	27	44	(38.0)	0.349	0.219
	−/1*60	20	26	(43.5)		
	1*60/1*60	4	2	(66.7)		
*UGT1A7* (387T>G)	387T/T	9	32	(22.0)	0.005	0.002
	387T/G	34	35	(49.3)		
	387G/G	8	5	(61.5)		
*UGT1A7* (622T>C)	622T/T	18	52	(25.7)	<0.001	<0.001
	622T/C	31	17	(64.6)		
	622C/C	2	3	(40.0)		
UGT1A9*1b	9*1b/9*1b	9	34	(20.9)	0.003	0.001
	−/9*1b	34	33	(50.7)		
	−*/*−	8	5	(61.5)		
Haplotypes						
*Hp-I*	0^a^	12	6	(66.7)	0.002	<0.001
	1^a^	32	37	(46.4)		
	2^a^	7	29	(19.4)		
*Hp-II*	0^a^	27	59	(31.4)	0.001	<0.001
	1^a^	22	13	(62.9)		
	2^a^	2	0	(100.0)		
*Hp-III*	0^a^	38	53	(41.8)	0.517	0.900
	1^a^	12	19	(38.7)		
	2^a^	1	0	(100.0)		
Clinical features						
Gender	Male	31	47	(39.7)	0.705	-
	Female	20	25	(44.4)		
Age	≤60	15	35	(30.0)	0.027	-
	>60	36	37	(49.3)		

a Number of alleles carried by the patient.

CA, Cochran-Armitage trend test.

**Table V:** Predicitive performance for irinotecan toxicity by the genotype combinations.

	Training (n=73)		Validation (n=50)	
		
	n	(%)	n	(%)
Matched with the combination^a^	62/73	(84.9)	43/50	(86.0)
Accuracy in applied patients	52/62	(83.9)	31/43	(72.1)
Positive predictive value^b^	19/22	(86.4)	7/10	(70.0)
Negative predictive value^b^	33/40	(82.5)	24/33	(72.7)
Accuracy	52/73	(71.2)	31/50	(62.0)
Sensitivity	19/34	(55.9)	7/17	(41.2)
Specificity	33/39	(84.6)	24/33	(72.7)

a The combination consists of 8 factors; 6 genotypes [UGT1A1*6, UGT1A1*28, UGT1A1*60, *UGT1A7* (387T>G), *UGT1A7* (622T>C) and UGT1A9*1b], gender and age.

b Prediction of severe toxicity is positive and prediction of no severe toxicity is negative.

**Table VI:** Associations between UGT1A genotypes/haplotypes and irinotecan toxicity in training and validation sub-populations.

	Training (n=73)						Validation (n=50)					
	Toxicity			Fisher’s exact test			Toxicity			Fisher’s exact test		
				
	Yes	No	(% of yes)	OR	(95% CI)	p-value	Yes	No	(% of yes)	OR	(95% CI)	p-value
Haplotypes												
*Hp-I* (+/+)	5	15	(25.0)	1^c^			2	14	(12.5)	1^c^		
*Hp-I* (−/−, −/+)	29	24	(54.7)	3.63	(1.15–11.42)	0.035	15	19	(44.1)	5.53	(1.08–28.18)	0.053
*Hp-II* (+/+,−/+)	16	8	(66.7)	0.64	(1.60–22.48)	0.008	8	5	(61.5)	11.20	(1.75–71.64)	0.016
The predicition system^a^												
Negative for toxicity	7	33	(17.5)	0.64	(0.17–2.34)	0.511	9	24	(27.3)	2.63	(0.50–13.92)	0.300
Positive for toxicity	19	3	(86.4)	8.00	(1.51–42.45)	0.021	7	3	(70.0)	16.33	(2.20–121.43)	0.009
Not matched^b^	8	3	(72.7)				1	6	(14.3)			
Genotypes												
UGT1A1*6 (+/+,−/+)	16	9	(64.0)	5.33	(1.45–19.58)	0.016	8	6	(57.1)	9.33	(1.51–57.65)	0.019
UGT1A1*28 (−/+)	7	7	(50.0)	3.00	(0.70–12.88)	0.163	4	2	(66.7)	14.00	(1.47–133.23)	0.025
UGT1A1*60 (+/+,−/+)	18	15	(54.5)	3.60	(1.06–12.22)	0.048	6	13	(31.6)	3.23	(0.55–18.96)	0.244
*UGT1A7* (387G/G, T/G)	27	21	(56.3)	3.86	(1.21–12.33)	0.032	15	19	(44.1)	5.53	(1.08–28.18)	0.053
*UGT1A7* (622C/C, T/C)	20	13	(60.6)	4.62	(1.35–15.78)	0.022	13	7	(65.0)	13.00	(2.27–74.32)	0.002
UGT1A9*1b (−/−, −/+)	27	20	(57.4)	4.05	(1.26–12.99)	0.018	15	18	(45.5)	5.83	(1.14–29.84)	0.028

a The prediction system consisted of the combinations of 8 factors (6 genotypes, gender and age).

b In training and validation populations, 9/73 (12.3%) and 7/50 (14.0%) patients were not matched with the combinations of the prediction system.

c Reference category.
